# Staged minimally invasive management of iatrogenic biliary-colonic fistula following laparoscopic cholecystectomy

**DOI:** 10.1093/gastro/goag096

**Published:** 2026-10-10

**Authors:** Xingzhou Zhang, Luyao Yang, Ming Wei, Nan Zhang

**Affiliations:** Department of Gastrointestinal Surgery, Tianjin Nankai Hospital, Tianjin Medical University, Tianjin, 300100, P. R. China; Tianjin Key Laboratory of Acute Abdomen Disease Associated Organ Injury and ITCWM Repair, Tianjin, 300100, P. R. China; Institute of Integrative Medicine for Acute Abdominal Diseases, Tianjin, 300100, P. R. China; Department of Gastrointestinal Surgery, Tianjin Nankai Hospital, Tianjin Medical University, Tianjin, 300100, P. R. China; Tianjin Key Laboratory of Acute Abdomen Disease Associated Organ Injury and ITCWM Repair, Tianjin, 300100, P. R. China; Institute of Integrative Medicine for Acute Abdominal Diseases, Tianjin, 300100, P. R. China; Tianjin Key Laboratory of Acute Abdomen Disease Associated Organ Injury and ITCWM Repair, Tianjin, 300100, P. R. China; Institute of Integrative Medicine for Acute Abdominal Diseases, Tianjin, 300100, P. R. China; Department of Obstetrics and Gynecology, Tianjin Nankai Hospital, Tianjin Medical University, Tianjin, 300100, P.R. China; Department of Gastrointestinal Surgery, Tianjin Nankai Hospital, Tianjin Medical University, Tianjin, 300100, P. R. China; Tianjin Key Laboratory of Acute Abdomen Disease Associated Organ Injury and ITCWM Repair, Tianjin, 300100, P. R. China; Institute of Integrative Medicine for Acute Abdominal Diseases, Tianjin, 300100, P. R. China

## Introduction

Biliary-colonic fistula is a rare life-threatening complication of laparoscopic cholecystectomy (LC), which typically results from unrecognized common hepatic duct injury [1, 2]. In contrast to spontaneous fistulas, iatrogenically induced fistulas are associated with a high risk of septic conditions. This paper reported a case successfully treated via a staged nonoperative strategy combining percutaneous double-lumen sump drain irrigation, endoscopic retrograde cholangiopancreatography (ERCP)-guided biliary diversion, and subsequent fistula occlusion. This approach controlled infection and promoted fistula tract narrowing, which serves as an effective alternative to conventional surgical repair for post-LC biliary-colonic fistula and avoids high-risk emergency laparotomy and stoma formation.

## Case report

A 60-year-old male patient was admitted with a 2-month history of right-upper-quadrant pain worsening over 3 days. The patient was diagnosed with acute calculous cholecystitis, with a 1.2-cm stone impacted at the neck of the gallbladder and severe pericholecystic adhesions. The patient subsequently underwent LC under general anesthesia. Intraoperatively, dense adhesions between the gallbladder, hepatic flexure of the colon, and liver bed were seen (Fig. 1A–D). During adhesiolysis, a partial laceration (∼3 mm) of the common hepatic duct (CHD) wall (1 cm below the hilum) was identified and primarily repaired by using 5–0 PDS-II sutures. A 10-Fr closed-suction drain was placed subhepatically. The patient developed persistent bile leakage and fever postoperatively.

**Figure 1:**
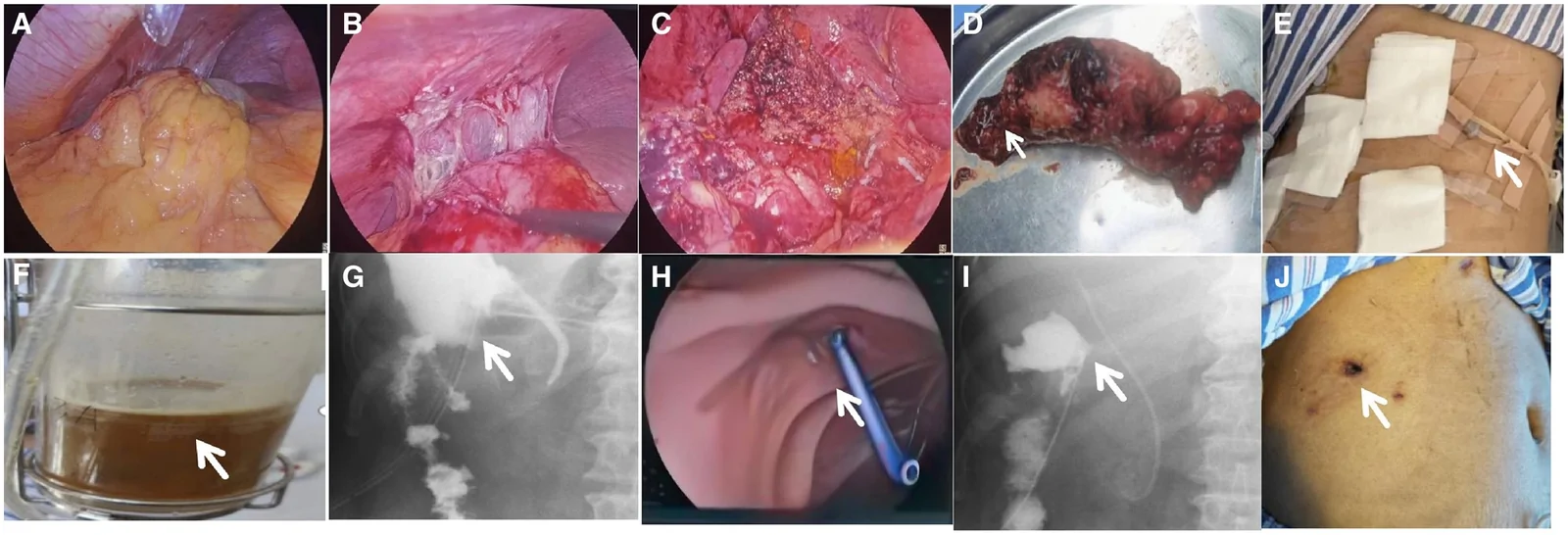
Intraoperative laparoscopic findings, imaging, and interventions. (A) Dense adhesions between gallbladder, hepatic flexure, and omentum. (B) Adhesions to lateral abdominal wall and liver bed. (C) Inflammatory hyperemia and edema with blurred boundaries. (D) Gangrenous gallbladder with impacted stone (white arrow). (E) Double-lumen sump drain placement (white arrow). (F) Serosanguineous drainage. (G) Fistulography showing biliary-colonic communication (white arrow). (H) A 15-cm 8 Fr plastic biliary stent was placed transpapillarily to divert bile flow internally and reduce fistula output (white arrow). (I) Fistulography showing closed biliary fistula and residual colonic tract (white arrow). (J) The colonic-fistula closure was successfully sealed (white arrow).

A stepwise nonoperative protocol was implemented:

Phase 1: Infection control and drainage (postoperative days 1–28). On postoperative day 4, the patient exhibited signs of localized peritonitis with serosanguineous discharge. Computed tomography (CT) revealed a fluid collection adjacent to the hepatic flexure. The original drain was replaced with a 14-Fr double-lumen sump drain for continuous negative-pressure suction (−80 mmHg) (Fig. 1E and F). Two weeks postoperatively, contrast fistulography confirmed a communication between the CHD suture site and the hepatic flexure of the colon (Fig. 1G). Ultrasound-guided puncture yielded purulent fluid (*Escherichia coli*-positive). Continuous negative-pressure irrigation with physiological saline was performed via the double-lumen drain to clear debris and control local infection.

Phase 2: Internal biliary diversion (postoperative days 29–64). After the local infection was effectively controlled, ERCP was performed. Intraoperative cholangiography confirmed the presence of bile leakage at the repair site. A 15-cm 8-Fr plastic biliary stent was placed transpapillarily to divert bile flow internally and reduce fistula output (Fig. 1H). After ERCP, continuous negative-pressure irrigation via a double-lumen drainage tube was maintained; the external drainage volume decreased significantly (<50 mL/day).

Phase 3: Tract maturation and delayed closure (postoperative days 65–84). At 65 days, the patient developed transient jaundice due to biliary sludge, managed by stent exchange (two stents placed). By 76 days postoperatively, fistulography confirmed that the biliary segment of the fistula had healed completely, with contrast agent only accumulating in the narrowed colonic-fistula tract (diameter < 0.5 cm) (Fig. 1I). Continuous negative-pressure irrigation via a double-lumen drainage tube was maintained. When the daily drainage volume decreased to <10 mL, the patient underwent the sealing of the colonic tubular fistula using bioprotein glue (Fig. 1J). After 1 week of observation, no liquid was found to be leaking from the colonic fistula. The patient recovered uneventfully and was discharged on day 84. At the 6-month follow-up, abdominal CT confirmed complete resolution with no fluid collection. The biliary stents were removed endoscopically without complications.

## Discussion

Iatrogenic biliary-colonic fistula is a catastrophic complication in which bile leakage erodes into the colon, creating a conduit for enteric bacteria to cause severe intra-abdominal infections. Isolated biliary erosion is unlikely to form a mature fistula within only 2 weeks after cholecystectomy. Unrecognized electrothermal injury or accidental mechanical trauma during a difficult adhesiolysis is regarded as the primary initiating factor for fistulization.

Historically, conventional open surgery has been the standard of care for biliary-colonic fistulas. Consequently, once diagnosed, definitive one-stage surgical repair is recommended for patients who are fit for the procedure [1]. Nevertheless, traditional surgical approaches entail significant drawbacks. Ojemolon noted that, given the high prevalence of multiple comorbidities (such as cardiovascular disease and diabetes) in ≤80% of patients with biliary-colonic fistulas, the morbidity and mortality rates associated with open surgery can be as high as 12%–27% [2].

The optimal management strategy is debated. Early surgical repair (e.g. T-tube drainage plus colostomy) is often associated with high morbidity in the setting of active inflammation and dense adhesions [3]. Conversely, pure conservative management often fails due to persistent high-output leakage and inadequate source control. Endoscopic management combining ERCP and colonoscopy also presents significant limitations, particularly with a high failure rate for large fistulas (>1 cm). To address these challenges, our staged nonoperative strategy addressed these challenges through a logical sequence: (i) aggressive source control: the use of a double-lumen sump drain (Leishi drain) allowed continuous negative-pressure irrigation, which mechanically cleared necrotic tissue and purulent debris, converting a complex abscess into a manageable sinus tract; (ii) physiological diversion: ERCP-guided stenting reduced intrabiliary pressure and diverted bile flow away from the fistula, removing the primary stimulus preventing healing [4]; (iii) delayed definitive repair: after fistulous tract narrowing, decreased daily fistula output, and complete infection control, definitive fistula sealing was performed with biological fibrin glue instead of surgical repair, which effectively reduced the risk of persistent leakage and obviated the need for stoma construction.

Conventional single-stage treatments for post-LC biliary-colonic fistulas are limited by insufficient infection control; our three-stage strategy combining dual-lumen irrigation, biliary stenting, and delayed fibrin-glue repair effectively optimizes the local fistula environment, achieving a higher healing rate and superior clinical outcomes compared with traditional single-modality therapies. This case supports shifting from early reoperation to endoscopic/percutaneous strategies [4, 5]. Our addition of continuous double-lumen drain irrigation uniquely manages the heavy bacterial load typical of biliary-colonic fistulas. While spontaneous colonic closure is rare [6], this staged approach successfully facilitated a minimally invasive final repair.

## Supplementary Material

goag096_Supplementary_Data
