# Supplementary material for: Staged minimally invasive management of iatrogenic biliary-colonic fistula following laparoscopic cholecystectomy
Source: Gastroenterol Rep (Oxf). 2026 Oct 10;14:goag096. doi: 10.1093/gastro/goag096 (PMC13653369; doi:10.1093/gastro/goag096)
Supplement: Supplementary file 1 [file goag096_supplementary_data.docx]

**Timelone**
